# Workplace violence and self-reported physical and mental health: a national cross-sectional study in Lebanon

**DOI:** 10.7189/jogh.16.04030

**Published:** 2026-02-27

**Authors:** Hazar Shamas, Ghada E Saad, Myriam Dagher, Rita Itani, Ali Abboud, Stephen J McCall, WOMENA Study Group, Jocelyn DeJong, Hala Ghattas, Nisreen Salti, Sasha Fahme, Serena Canaan, Malak Ghezzawi, Hazar Shamas, Ghada E Saad, Myriam Dagher, Rita Itani, Ali Abboud, Stephen J McCall

**Affiliations:** 1 Center for Research on Population and Health, Faculty of Health Sciences, American University of Beirut, Beirut, Lebanon; 2 Department of Economics, American University of Beirut, Beirut, Lebanon

## Abstract

**Background:**

Few studies have investigated the prevalence of workplace violence (WPV) – defined as any physical or psychological violence experienced in the workplace – in the Middle East and North Africa (MENA) region. We aimed to examine the determinants of WPV and its association with self-reported physical and mental health among employed adults residing in Lebanon.

**Methods:**

This was a national cross-sectional study among working-age residents of Lebanon recruited through random digit dialling. Data were collected from January to July 2024. The main exposure of interest was WPV, defined as having experienced at least one incident of physical or psychological violence in the past six months. We measured three outcomes – depressive symptoms, anxiety symptoms, and poor physical health – using the Patient Health Questionnaire-9, Generalized Anxiety Disorder-7, and Patient-Reported Outcomes Measurement Information System Global Health 1.2 scales, respectively. Adjusted logistic regression models, accounting for covariates identified through directed acyclic graphs, estimated the association between WPV and each outcome, separately.

**Results:**

We enrolled 3076 employed participants with a median age of 37 years (interquartile range = 28–46), of whom 30.5% had completed college or postgraduate education and 24.9% were non-Lebanese. Almost 16% of participants experienced at least one form of WPV. Moreover, 33.4%, 25.5%, and 75.7% experienced depressive symptoms, anxiety symptoms, and reported poor physical health, respectively. Being exposed to WPV increased the odds of depressive symptoms (adjusted odds ratio (aOR) = 3.00; 95% confidence interval (CI) = 2.40–3.70), anxiety symptoms (aOR = 3.01; 95% CI = 2.41–3.72), and poor physical health (aOR = 2.82; 95% CI = 2.04–3.98) after adjusting for age, sex, education, nationality, marital status, urbanicity, and job sector.

**Conclusions:**

Our findings highlight the extent and effect of WPV among workers in Lebanon and the urgent need to address the matter. Findings offer a basis for developing targeted interventions aimed to support vulnerable workers.

Workplace violence (WPV), defined as any physical or psychological violence experienced in the workplace, can take various forms, with workplace physical violence including sexual assault, hitting, spitting, shoving, and use of weapons [1,2]. Workplace psychological violence manifests as verbal threats, bullying, manipulation, micromanagement, and isolation. WPV is a form of stigmatisation, discrimination, and inequality [2]. Globally, almost 23% of employed adults experience at least one form of violence at work, most of which is psychological [3]. Studies have shown that workers experiencing WPV are at a higher risk of developing anxiety symptoms, depressive symptoms, and overall fatigue [4,5]. Furthermore, WPV has been associated with adverse outcomes such as absence due to sickness, sleep-related problems, stress, burnout, binge drinking, social phobia, suicide ideation, suicide, and poor physical and mental health [6–10]. Most research examining WPV has been conducted in high-income countries and targeted the formal sector, particularly the healthcare sector [6,7,9,10]. Studies in the Middle East and North Africa (MENA) region, meanwhile, primarily focused on the impact of WPV on the mental and physical health of workers in healthcare settings only and found that psychological violence was more prevalent than physical violence [11–13].

Lebanon is a low-middle-income country (LMIC) situated in the MENA region [14]. It has been impacted by political instability and economic crises, with much of the population consequently enduring financial hardship [15] – a factor known to be associated with a higher prevalence of WPV [16]. As in the MENA region in general, studies investigating WPV in Lebanon were limited to the healthcare sector and focused on healthcare workers, doctors, and nurses. Their findings indicated a high prevalence of WPV among nurses specifically, with 62% reporting verbal abuse and 10% reporting physical violence [17,18].

In addition, Lebanon hosts the highest number of refugees per capita in the world [19], amounting to a quarter of its population [20]. Most of these refugees are Syrians, many of whom lack residency permits, are informally employed (*i.e.* without written contracts), are discriminated against, have no legal protection, and are subjected to workplace exploitation [4,20,21]. Limited studies on WPV in low-resource settings have been conducted at the national level, particularly in settings with a large informal labour sector and a large refugee population. Thus, we aimed to examine the determinants of WPV and its association with self-reported physical and mental health among Lebanese and non-Lebanese employed adults residing in Lebanon.

## METHODS

### Study design and setting

This was a national cross-sectional telephone survey study in Lebanon, nestled within a larger project titled: ‘Identifying opportunities to improve the lived experience and health of working women in the MENA: from COVID to recovery’ (WOMENA). We reported its findings per the STROBE guidelines [22].

### Sampling and study population

We recruited residents in Lebanon through random digit dialling between January and July 2024. Numbers dialled were constructed within an 11-digit frame used by mobile phones in Lebanon. The first three numbers consisted of +961 (international country calling code for Lebanon), followed by two digits implying the country’s mobile network operators (*i.e.* one pair of the following: 03, 70, 71, 76, 78, 79, 81), followed by a randomly generated set of six numbers. We called each potential participant a maximum of two times if the call was not picked up the first time. If respondents were unable to complete the survey, we scheduled follow-up calls for a convenient time. Calls were made between 9:00 and 19:00 Beirut local time (GMT +2 in winter) during weekdays and Saturdays. Calls outside these hours were conducted only if participants requested to reschedule.

We determined eligibility using a screening questionnaire, where we invited residents in Lebanon aged 19–64 years to participate in the study (Figure S1 in the **Online Supplementary Document**). While the WOMENA study enrolled both employed and unemployed residents, we restricted our sample to the former group only. Women were oversampled to ensure a sufficient number of employed men and women.

We obtained informed oral consent from all participants prior to their enrolment. A strategic monitoring plan with standardised data quality procedures was adopted while collecting data. Specifically, we monitored data collection weekly to detect and mitigate systematic errors and missing data on time. We also recorded five percent of the surveys and compared them with the actual collected data to identify any data errors. We calculated the error rate, computed as the number of identified errors out of the total number of answered questions, to be 0.6%.

### Data sources

The survey incorporated existing validated questionnaire modules, scales, and community-identified priorities adapted to the Lebanese context: the Patient Health Questionnaire-9 (PHQ-9), Generalized Anxiety Disorder-7 (GAD-7), and Patient-Reported Outcomes Measurement Information System Global Health 1.2 (PROMIS GBH). The survey was initially developed in English and then translated to Arabic.

Trained data collectors interviewed the participants in their mother tongue and recorded their data through the SurveyCTO software. Individuals who consented and were eligible for participation answered questions regarding their sociodemographics, employment history and characteristics, physical health, anxiety and depressive symptoms.

### Main exposure

The main exposure in this study was WPV, determined according to responses to the following question extracted from the International Labour Organization Gallup [23]: ‘During the past six months, have you ever experienced at least one of the following physical violence and/or harassment at work, such as hitting, restraining, or spitting; psychological violence and/or harassment at work, such as insults, threats, bullying, or intimidation?’

### Outcomes of interest

Three outcomes were explored in this study: depressive symptoms, anxiety symptoms, and self-reported poor physical health. We measured depressive symptoms through the PHQ-9 [24], a nine-item questionnaire that has been validated in Arabic [25]. Its total summative score ranges from 0 to 27, with each item scored from 0 (not at all) to 3 (nearly every day), whereby a total score of 10 or more indicates the presence of depressive symptoms.

We measured anxiety symptoms through the GAD-7 [26], a seven-item questionnaire that has been validated in Arabic [27]. Its total summative score ranges from 0 to 21, with each item having a score of 0 (not at all) to 3 (nearly every day), whereby a total score of 10 or more indicates the presence of anxiety symptoms.

We measured self-reported poor physical health using the reliable PROMIS GBH 1.2 scale [28], which includes four questions about physical health. The score for each question ranges from 1 (poor physical health) to 5 (excellent physical health). We subsequently summed up the question scores and dichotomised them into good physical health, defined as the highest 25th percentile of scores, and poor physical health, defined as the lowest 75th percentile.

The PHQ-9, GAD-7, and PROMIS GBH 1.2 scales had excellent reliability in this population with a Cronbach’s alpha of 0.80, 0.85, and 0.80 respectively.

### Confounders

We identified confounders using directed acyclic graphs constructed using the DAGitty software [29,30] based on a validated methodology, where causal paths between the main exposure (WPV) and the three outcomes: depressive symptoms (PHQ-9 score ≥10), anxiety symptoms (GAD-7 score ≥10), and poor physical health (PROMIS GBH score <75th percentile) were identified (Figures S2–4 in the **Online Supplementary Document**) [30]. Through this approach, we identified age, sex, education, nationality, marital status, urbanicity, and job sector to be confounders in these associations.

We assessed urbanicity using the single-item self-report measure (SIDU): ‘Please indicate how urban your living environment is on a seven-point scale from 1 (not urban at all) to 7 (very urban).’ The responses were then dichotomised, where a score of 6 and more implied extremely urbanised [31]. Participants were attributed to one job sector depending on their work. The job sectors included: government or non-governmental organisations (NGOs) (*i.e.* government institutions, state-owned enterprises, embassies or international organisations, political parties); private businesses; private household jobs (*i.e.* individuals employed full-time in a private household such as domestic workers or private drivers, private cleaners, private chefs); freelance or informal sector jobs (*i.e.* self-employed individuals or individuals working independently for multiple employers or clients, typically without a formal employment contract or guaranteed benefits).

### Statistical analysis

We identified determinants of WPV and their association with the outcomes based on existing literature. We presented the frequency of participants reporting each outcome and their weighted percentages, as well as unadjusted weighted odds ratios (ORs) and 95% confidence intervals (CIs). We calculated unadjusted and adjusted absolute prevalence differences (APDs) and their 95% CIs for the three outcomes among participants who experienced WPV [32]. To account for the study design that aimed to have equal allocation of employed and unemployed males and females, we calculated sampling and post-calibration weights based on nationality differences to allow for national estimates.

We ran separate logistic regressions to estimate the association between WPV and each of the outcomes: depressive symptoms, anxiety symptoms, and self-reported poor physical health. We analysed missing values using Little’s test of missing completely at random. The maximum number of missing values in a variable was 1.5%, and complete case analyses was implemented [33,34].

We performed all analyses using Stata, version 18 (StataCorp LLC, College Station, Texas, USA). Coefficient estimates with *P*-values <0.05 were considered statistically significant.

## RESULTS

We placed 97 608 calls using random digit dialling, to which we received 32 411 (33.2%) responses, while the rest were unreachable. Among those who responded, 23 495 (75.5%) did not consent, 1534 (4.8%) were ineligible, and 10 (0.03%) were previously contacted by the team on their second phone number, leaving 7372 (22.7%) eligible participants. A total of 4725 participants completed the survey, out of whom 3076 (65.1%) were employed constituting the unit of analyses in this study (Figure S1 in the **Online Supplementary Document**).

The median age of the 3076 participants was 37 years (interquartile range = 28−46), 889 (30.5%) completed college or postgraduate education, 2130 (68.6%) were married or engaged, and 1111 (24.9%) were non-Lebanese. A total of 518 (16%) participants experienced at least one form of WPV, of whom 36 (7%) experienced physical violence only, 374 (72.2%) experienced psychological violence only, and 108 (20.8%) experienced both. Psychological violence was the most prevalent form of WPV experienced among participants (n/N = 482/518, 93%).

**Figure 1:**
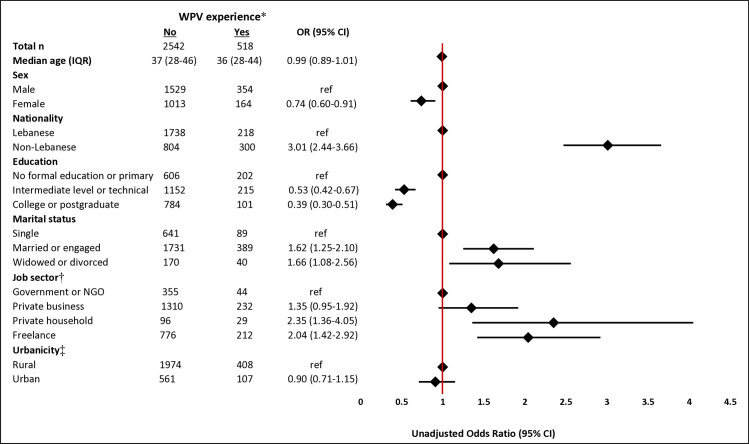
Unadjusted ORs of workplace violence stratified by sociodemographics. CI – confidence interval, IQR – interquartile range, NGO – non-governmental organisation, OR – odds ratio, ref – reference, WPV – workplace violence (not presented in this figure). *Participants reporting WPV: n = 3060. †Participants reporting job sector: n = 3054. ‡Participants reporting urbanicity: n = 3050.

Females had lower odds of experiencing WPV compared to males (OR = 0.74; 95% CI = 0.60–0.91). Similarly, those with intermediate level or technical education and those who completed college or postgraduate education had lower odds of experiencing WPV compared to those with no formal education or primary education only (OR = 0.53; 95% CI = 0.42–0.67) and (OR = 0.39; 95% CI = 0.30–0.51), respectively. The odds of experiencing WPV were higher among non-Lebanese compared to Lebanese individuals (OR = 3.01; 95% CI = 2.44–3.66), and among freelance workers compared to those who work in the government or NGOs (OR = 2.04; 95% CI = 1.42–2.92) (**Figure 1**) and compared to those who work in private business (OR = 1.50; 95% CI = 1.21–1.86).

The odds of having depressive symptoms were higher among non-Lebanese compared to Lebanese individuals (OR = 1.99; 95% CI = 1.70–2.32), those who are widowed or divorced compared to single (OR = 1.92; 95% CI = 1.38–2.67), and those who work in either private household (OR = 2.12; 95% CI = 1.37–3.27) or freelance (OR = 1.56; 95% CI = 1.20–2.02) compared to those who work in the government or NGOs. The odds of having depressive symptoms were lower among those who completed college or postgraduate education compared to those with no formal education or primary education only (OR = 0.43; 95% CI = 0.35–0.53) (**Table 1**).

**Table 1:** Characteristics of participants and their association with depressive symptoms*

	Total (n = 3036, 100%)	PHQ-9 score <10 (n = 1956, 66.6%)	PHQ-9 score ≥10 (n = 1080, 33.4%)†	Unadjusted OR (95% CI)
**Median age (IQR)**	37 (29–47)	38 (29–47)	37 (29–47)	0.99 (0.99–1.01)
**Sex**				
Male	1865 (67.4)	1222 (68.2)	643 (65.8)	ref
Female	1171 (32.6)	734 (31.8)	437 (34.2)	1.11 (0.95–1.30)
**Education**				
No formal education or primary	801 (24.5)	430 (20.6)	371 (32.3)	ref
Intermediate level or technical	1357 (45.1)	883 (45.4)	474 (44.5)	0.62 (0.51–0.75)
College or postgraduate	878 (30.4)	643 (34)	235 (23.2)	0.43 (0.35–0.53)
**Nationality**				
Lebanese	1941 (75)	1366 (79)	575 (66.1)	ref
Non-Lebanese	1095 (25)	590 (21)	505 (33.9)	1.99 (1.70–2.32)
**Marital status**				
Single	721 (25.1)	516 (27.4)	205 (20.6)	ref
Married or engaged	2105 (68.7)	1325 (67.2)	780 (71.6)	1.41 (1.17–1.71)
Widowed or divorced	210 (6.2)	115 (5.4)	95 (7.8)	1.92 (1.38–2.67)
**Urbanicity**				
Rural	2336 (79.2)	1499 (78.6)	837 (80.4)	ref
Urban	664 (20.8)	433 (21.4)	231 (19.6)	0.89 (0.73–1.07)
Missing	36	24	12	
**Job sector**				
Government or NGO	392 (14.4)	276 (15.4)	116 (12.5)	ref
Private business	1536 (51)	1037 (53.2)	499 (46.6)	1.07 (0.84–1.38)
Private household	125 (3.3)	68 (2.7)	57 (4.6)	2.12 (1.37–3.27)
Freelance	977 (31.3)	570 (28.7)	407 (36.4)	1.56 (1.20–2.02)
Missing	6	5	1	

CI – confidence interval, IQR – interquartile range, NGO – non-governmental organisation, OR – odds ratio, PHQ-9 – Patient Health Questionnaire-9, ref – reference

*Values are presented as n (%) unless specified otherwise.

†PHQ-9 score ≥10 indicates having depressive symptoms.

Similarly, the odds of having anxiety symptoms were higher among non-Lebanese compared to Lebanese (OR = 1.60; 95% CI = 1.36–1.89), widowed or divorced compared to single (OR = 1.93; 95% CI = 1.36–2.74), and those working in freelance compared to those working in the government or NGOs (OR = 1.41; 95% CI = 1.07–1.84) (Table S1 in the **Online Supplementary Document**).

Females, non-Lebanese, widowed or divorced, and freelance workers had higher odds of reporting poor physical health (OR = 2.27; 95% CI = 1.86–2.76), (OR = 1.68; 95% CI = 1.39–2.04), (OR = 2.67; 95% CI = 1.69–4.06), (OR = 1.65; 95% CI = 1.24–2.20) compared to males, Lebanese, singles, and those working in the government or NGOs, respectively (**Table 2**).

**Table 2:** Characteristics of participants and their association with self-reported poor physical health*

	Total (n = 3047, 100%)	PROMIS GBH score ≥75th percentile ( n = 673, 24.3%)	PROMIS GBH score <75th percentile (n = 2374, 75.7%)†	Unadjusted OR (95% CI)
**Median age (IQR)**	38 (29–47)	35 (27–45)	38 (30–47)	1.01 (1.00–1.02)
**Sex**				
Male	1872 (67.3)	510 (79.7)	1362 (63.4)	ref
Female	1175 (32.7)	163 (20.3)	1012 (36.6)	2.27 (1.86–2.76)
**Education**				
No formal education or primary	798 (24.2)	132 (17.5)	666 (26.4)	ref
Intermediate level or technical	1365 (45.2)	300 (44.8)	1065 (45.3)	0.67 (0.53–0.85)
College or postgraduate	884 (30.6)	241 (37.7)	643 (28.3)	0.49 (0.38–0.63)
**Nationality**				
Lebanese	1941 (74.8)	490 (81)	1451 (72.6)	ref
Non-Lebanese	1106 (25.2)	183 (19)	923 (27.4)	1.68 (1.39–2.04)
**Marital status**				
Single	727 (25.2)	233 (34.8)	494 (22.1)	ref
Married or engaged	2112 (68.6)	411 (61.1)	1701 (71.1)	1.83 (1.50–2.22)
Widowed or divorced	208 (6.2)	29 (4.1)	179 (6.8)	2.67 (1.69–4.06)
**Urbanicity**				
Rural	2346 (79.1)	513 (78.1)	1833 (79.4)	ref
Urban	668 (20.9)	153 (21.9)	515 (20.6)	0.92 (0.74–1.14)
Missing	33	7	26	
**Job sector**				
Government or NGO	396 (14.6)	102 (17.1)	294 (13.8)	ref
Private business	1538 (50.9)	382 (56.6)	1156 (49)	1.07 (0.83–1.39)
Private household	124 (3.2)	12 (1.3)	112 (3.9)	3.6 (1.85–7.00)
Freelance	983 (31.3)	175 (25)	808 (33.3)	1.65 (1.24–2.20)
Missing	6	2	4	

CI – confidence interval, IQR – interquartile range, NGO – non-governmental organisation, OR – odds ratio, PROMIS GBH – Patient-Reported Outcomes Measurement Information System Global Health, ref – reference

*Values are presented as n (%) unless specified otherwise.

†PROMIS GBH score <75th percentile indicates reporting poor physical health.

**Figure 2:**
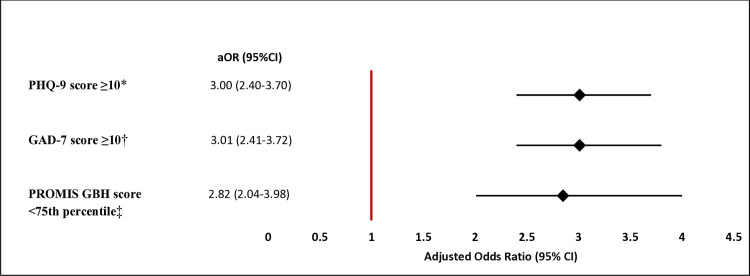
Adjusted ORs of the relationship between workplace violence and each of depressive symptoms, anxiety symptoms, and poor physical health. aOR – adjusted odds ratio, CI – confidence interval, GAD-7 – Generalized Anxiety Disorder-7, PHQ-9 – Patient Health Questionnaire-9, PROMIS GBH – Patient-Reported Outcomes Measurement Information System Global Health. *PHQ-9 score ≥10 indicates having depressive symptoms. †GAD-7 score ≥10 indicates having anxiety symptoms. ‡PROMIS GBH score <75th percentile indicates reporting poor physical health.

Accounting for age, sex, education, nationality, marital status, urbanicity, and job sector, the odds of having depressive symptoms, anxiety symptoms, and reporting poor physical health were almost three times higher among individuals who experienced WPV compared to those who did not, with adjusted odds ratios of 3.00 (95% CI = 2.40–3.70), 3.01 (95% CI = 2.41–3.72), and 2.82 (95% CI = 2.04–3.98), respectively (**Figure 2**). Moreover, accounting for the same factors, the prevalence of having depressive symptoms, anxiety symptoms, and reporting poor physical health, among participants exposed to WPV, was higher by 40 percentage points (adjusted APD (aAPD) = 0.40; 95% CI = 0.26–0.53), 28 percentage points (aAPD = 0.28; 95% CI = 0.14–0.41), and 15 percentage points (aAPD = 0.15; 95% CI = 0.04–0.26), respectively, compared with participants who were not exposed to WPV (Table S2 in the **Online Supplementary Document**).

## DISCUSSION

Nearly one out of six employed adults in Lebanon experienced at least one form of WPV, with psychological violence being the most common. Furthermore, WPV was associated with almost triple odds of displaying depressive symptoms, anxiety symptoms, and reporting poor physical health in a nationally representative sample of working-aged adults in Lebanon. The odds of experiencing WPV were higher among males, those with no formal education or primary education only, non-Lebanese residents, and freelance workers.

In our study, the prevalence of WPV among employed adults in Lebanon was 16%, which is relatively high compared to other middle and high-income countries [10,35–37]. The national prevalence of WPV in most of those countries ranged from 4.7% to 20% [38]. This may be explained by the large number of freelance workers in Lebanon, most of whom belong to the informal sector in this nationally representative sample [39,40]. Previous research has shown that freelance workers are more prone to encounter WPV, as they lack protection and formal support systems such as a human resource department and insurance [4,21,41]. In addition, freelance workers are likely to have volatile job circumstances and are less prone to report or address incidents of WPV due to fear of losing income or future work opportunities [23,42]. Another explanation for the high prevalence of WPV in this study was the large proportion of non-nationals in Lebanon. Previous studies have shown WPV was experienced more among non-national workers compared to national workers, particularly among refugees or migrant workers [21,43]. Refugee or migrant populations often lack social network or legal protection that prevents discrimination in the workplace [21,38]. In addition, many refugees in Lebanon lack legal residency permits and only have oral contracts with their employers or no contract [20,21]. As such, refugees are primarily employed in the informal sectors, which places refugees at risk of exploitation and poor working conditions [21].

In 2020, Lebanon enacted law number 205 criminalising sexual harassment across different settings, including the workplace, regardless of nationality [44]. However, this law may not be strictly enforced in the workplace and, importantly, does not protect against physical or psychological violence [44,45]. Thus, future legislation and policies are required to include all forms of violence, and mechanisms should be established to enforce these policies at the institutional and organisational levels.

Associations between sex and WPV vary across research, depending on the occupational context or reporting practices [46]. We found that employed males in Lebanon had higher odds of WPV than females. Almost half of our male participants were non-nationals, as opposed to 25% of female participants, which may explain the higher odds of WPV among males. We did not find urbanicity to be associated with WPV. This is in contrast with other studies that measured WPV in urban settings [47,48] – a discrepancy that warrants further investigation.

In line with findings from multiple systematic and scoping reviews [6,9,46,49], WPV in our study was associated with depressive symptoms, anxiety symptoms, and poor physical health. This may be explained by the social consequences of experiencing WPV that manifest as social isolation, job dissatisfaction, burnout, and poor engagement, leading to reduced productivity, lack of motivation, lower self-esteem in the job, and poor mental health [49]. The impact of experiencing WPV varies by individual, but could be considered as a traumatic event that leads to feelings of fear, shame, chronic stress, helplessness, and loss of control, inducing depression and anxiety. Experiencing WPV was also associated with reporting poor physical health in our study, which may be a result of physical injuries from the violence, pain, back pain, headache or eye strain, overall fatigue, and possible disability [50–52].

This study has several limitations. It has a cross-sectional design, meaning temporality was not met, leaving a need to follow up this population in the future. We did not collect several potentially important variables, such as personality traits and prior trauma; as such, the estimated coefficients may be prone to residual confounding. Our outcomes of interest were self-reported, which could have led to misclassification bias. Additionally, we made over 97 000 calls and had 3076 employed respondents; individuals who responded to calls and consented to a lengthy survey might have differed systematically from non-responders, which may have either differentially or non-differentially affected the effect estimates. To minimise the impact of this bias, we calculated sampling weights that account for differences in the sample and the general population. Furthermore, we are confident in the quality of the collected data, as 5% of the recorded interviews were quality-checked, giving a 0.6% error rate.

This is one of the very few and largest nationally representative cross-sectional studies in the MENA region to examine the association of WPV with mental and physical health among employed individuals, especially those working within the informal sector. Its results show that experiencing any form of WPV, at least once, is associated with having depressive and anxiety symptoms, and reporting poor physical health. Future research should explore the intensity and frequency of WPV against health outcomes to understand whether a threshold effect or dose-response relationship exists.

Our findings also highlight the implications of experiencing WPV on employees’ physical and mental health, indicating a need to ensure a safe, secure, and productive work environment for employees in the informal and formal work sectors. As WPV is a multifactorial problem, it requires interventions at multiple levels: policy and legislative interventions (*e.g.* zero tolerance policy for WPV), organisational interventions (*e.g.* confidential reporting systems), and interventions that target behavioural change [18].

## CONCLUSIONS

Our study explores the extent of WPV among workers in Lebanon, its impact on their well-being, and the underlying determinants. Its results show that being a non-national, having no formal education or primary education only, and working in an unstable job increased the odds of WPV. Future research should evaluate appropriate multi-level interventions, including those that are policy and legislative, organisational, or behavioural levels. Our findings thus offer a basis for targeting such interventions that promote respect and tolerance between workers within the workplace.

## Additional material


Online Supplementary Document
