# Supplementary material for: Workplace violence and self-reported physical and mental health: a national cross-sectional study in Lebanon
Source: J Glob Health. 2026 Feb 27;16:04030. doi: 10.7189/jogh.16.04030 (PMC12945342; doi:10.7189/jogh.16.04030)
Supplement: Online Supplementary Document [file jogh-16-04030-s001.pdf]

Supplement to: Shamas H, Saad GE, Dagher M, Itani R, Abboud A, McCall SJ. Workplace violence and self-reported physical and mental health: a national cross-sectional study in Lebanon. J Glob Health. 2026;16:04030.

Table of Contents

Figure S1. Flow diagram representing the participants included in the study 3

Figure S2. DAG representing the association between workplace violence and depressive symptoms 4

Figure S3. DAG representing the association between workplace violence and anxiety symptoms 5

Figure S4. DAG representing the association between workplace violence and poor physical health........ 6

Table S1. Characteristics of participants and their association with anxiety symptoms* 7

Table S2. Unadjusted and adjusted absolute prevalence difference in mental and physical health by workplace violence 8

Figure S1. Flow diagram representing the participants included in the study

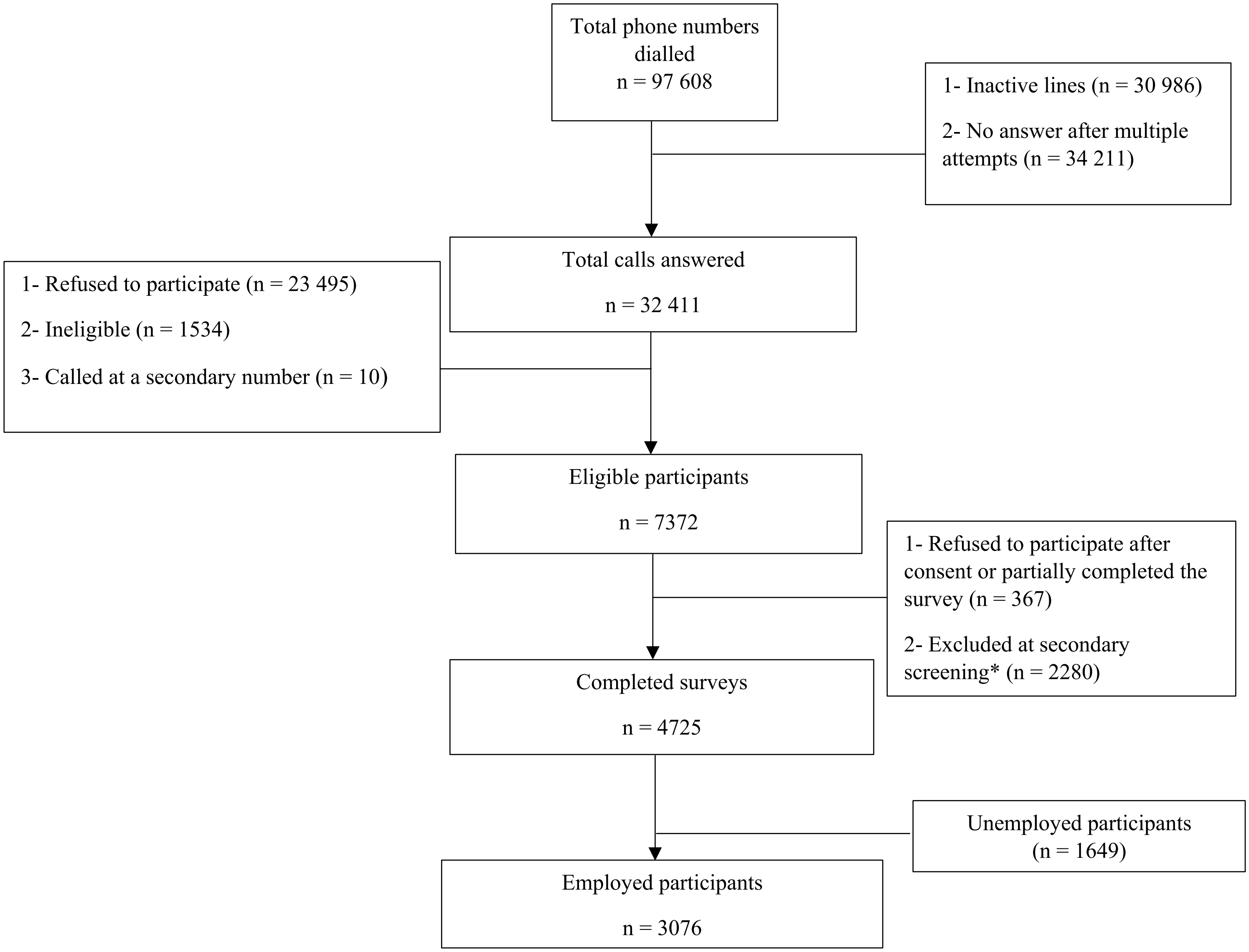

*At this stage, we further assessed participants' employment status and sex to enable oversampling of employed women in line with national estimates.

Figure S2. DAG representing the association between workplace violence and depressive symptoms

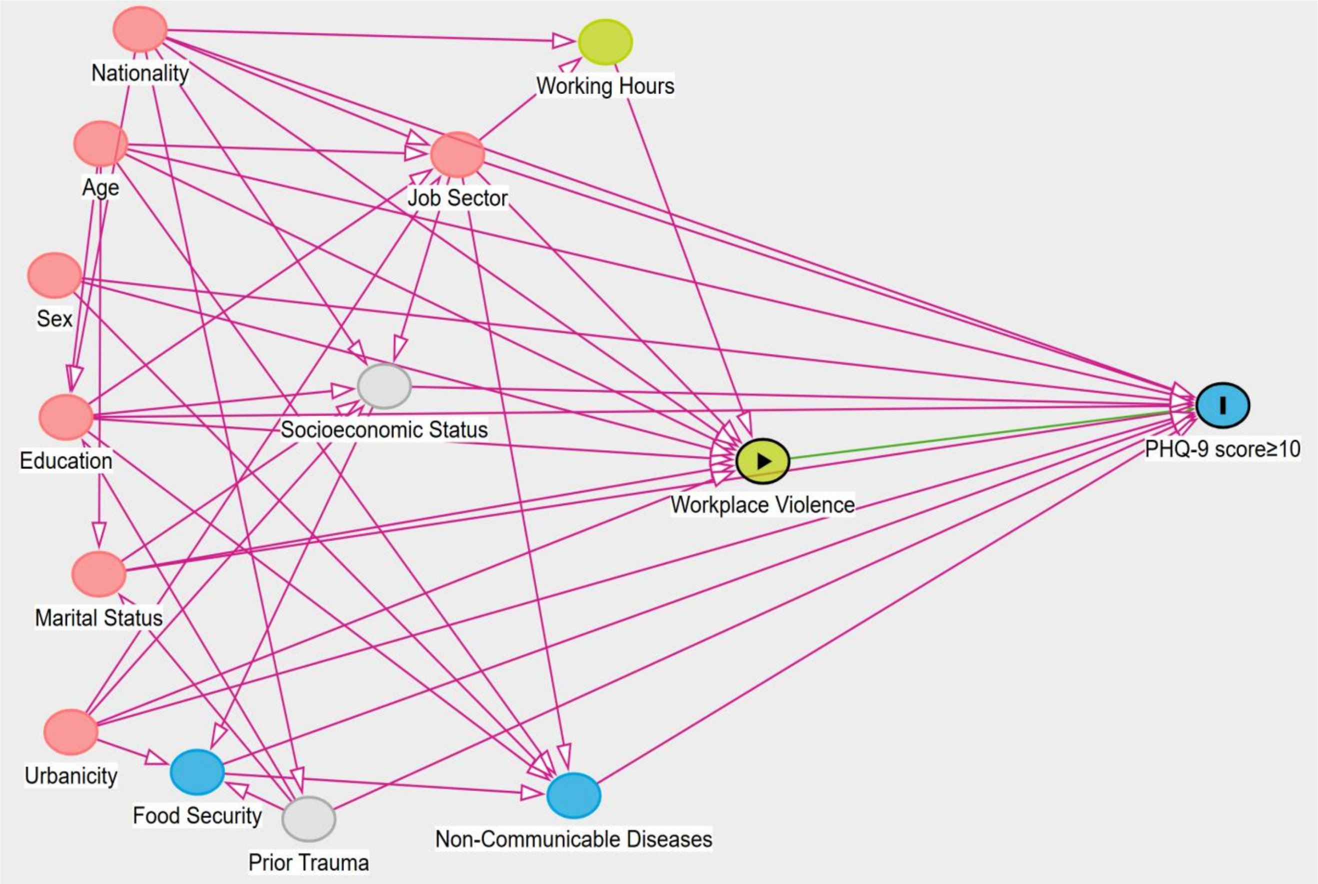

PHQ-9 − Patient Health Questionnaire-9, PHQ-9 score ≥10 indicates having depressive symptoms.

Figure S3. DAG representing the association between workplace violence and anxiety symptoms

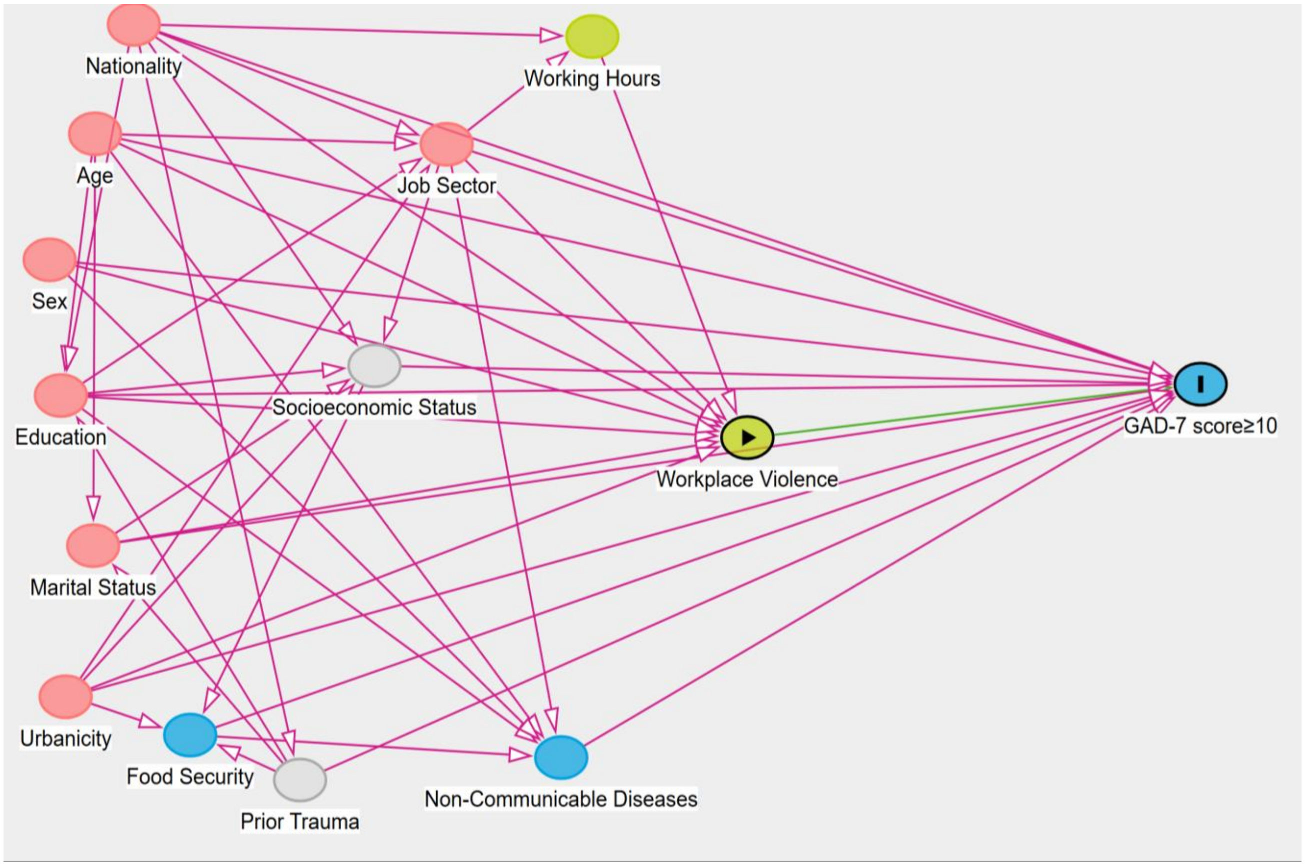

GAD-7 − Generalized Anxiety Disorder-7, GAD-7 score ≥10 indicates having anxiety symptoms.

Figure S4. DAG representing the association between workplace violence and poor physical health

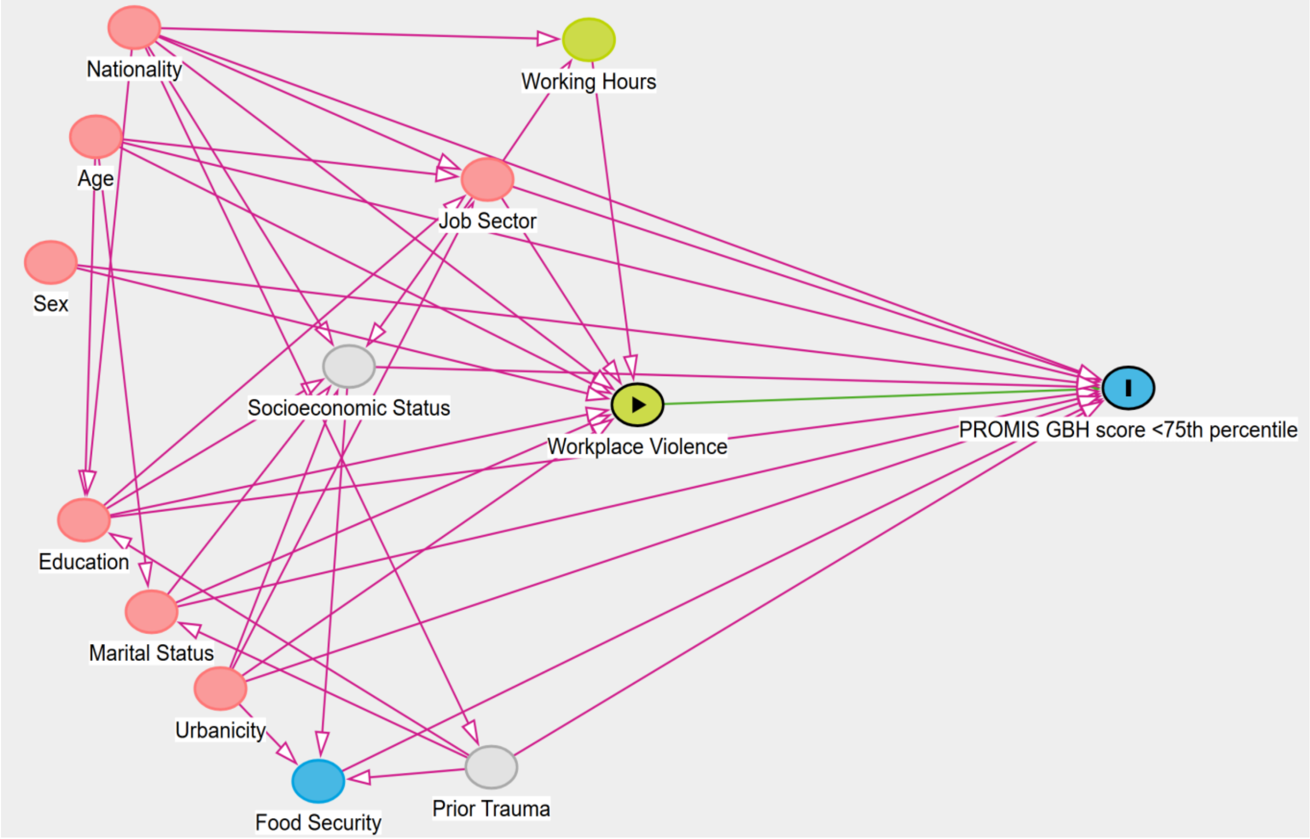

PROMIS GBH − Patient-Reported Outcomes Measurement Information System Global Health, PROMIS GBH score <75th percentile indicates reporting poor physical health.

Table S1. Characteristics of participants and their association with anxiety symptoms*

	Total		GAD-7 score <10		GAD-7 score ≥10†		Unadjusted OR (95% CI)
	**n = 3053**	**(100%)**	**n = 2228**	**(74.5%)**	**n = 825**	**(25.5%)**	
**Median age (IQR)**	38	(29−47)	37	(28−47)	38	(29−47)	1.01 (0.99−1.02)
**Sex**							
Male	1874	(67.3)	1403	(68.4)	471	(63.9)	ref
Female	1179	(32.7)	825	(31.6)	354	(36.1)	1.20 (1.03−1.44)
**Education**							
No formal education or primary	806	(24.5)	532	(22.4)	274	(30.8)	ref
Intermediate level or technical	1364	(45.1)	981	(44.2)	383	(47.6)	0.78 (0.64−0.95)
College or postgraduate	883	(30.4)	715	(33.4)	168	(21.6)	0.47 (0.37−0.59)
**Nationality**							
Lebanese	1955	(75)	1499	(77.5)	456	(68.2)	ref
Non-Lebanese	1098	(25)	729	(22.5)	369	(31.8)	1.60 (1.36−1.89)
**Marital status**							
Single	731	(25.3)	577	(27)	154	(20.2)	ref
Married or engaged	2113	(68.5)	1518	(67.4)	595	(71.7)	1.41 (1.14−1.74)
Widowed or divorced	209	(6.2)	133	(5.6)	76	(8.1)	1.93 (1.36−2.74)
**Urbanicity**							
Rural	2350	(79.2)	1716	(79.1)	634	(79.2)	ref
Urban	667	(20.8)	484	(20.9)	183	(20.8)	0.99 (0.81−1.21)
Missing	36		28		8		
**Job sector**							
Government or NGO	397	(14.5)	298	(14.7)	99	(13.8)	ref
Private business	1540	(50.9)	1173	(53.1)	367	(44.3)	0.89 (0.68−1.16)
Private household	125	(3.3)	89	(3.1)	36	(3.7)	1.28 (0.80−2.06)
Freelance	985	(31.3)	663	(29.1)	322	(38.2)	1.41 (1.07−1.84)
Missing	6		5		1		

CI − confidence interval, GAD-7 − Generalized Anxiety Disorder-7, IQR − interquartile range, NGO − non-governmental organisation, OR − odds ratio, ref − reference

*Values are presented as n (%) unless specified otherwise.

†GAD-7 score ≥10 indicates having anxiety symptoms.

Table S2. Unadjusted and adjusted absolute prevalence difference in mental and physical health by workplace violence

Outcomes	Unadjusted APD (95%CI)	Adjusted APD (95% CI)
**PHQ-9 score ≥10***	0.29 (0.24−0.34)	0.40 (0.26−0.53)
**GAD-7 score ≥10†**	0.59 (0.55−0.62)	0.28 (0.14−0.41)
**PROMIS GBH score <75th percentile‡**	0.16 (0.13−0.19)	0.15 (0.04−0.26)

APD − absolute prevalence difference, CI − confidence interval, GAD-7 − Generalized Anxiety Disorder-7, PHQ-9 − Patient Health Questionnaire-9, PROMIS GBH − Patient-Reported Outcomes Measurement Information System Global Health
*PHQ-9 score ≥10 indicates having depressive symptoms, †GAD-7 score ≥10 indicates having anxiety symptoms.
‡PROMIS GBH score <75th percentile indicates reporting poor physical health.
